# Ginsenoside Rd inhibits migration and invasion of tongue cancer cells through H19/miR-675-5p/CDH1 axis

**DOI:** 10.1590/1678-7757-2022-0144

**Published:** 2022-09-02

**Authors:** Lu CHANG, Dongxu WANG, Shaoning KAN, Ming HAO, Huimin LIU, Zhijing YANG, Qianyun XIA, Weiwei LIU

**Affiliations:** 1 Jilin University Hospital of Stomatology Department of Oral and Maxillofacial Surgery Changchun China Jilin University, Hospital of Stomatology, Department of Oral and Maxillofacial Surgery, Changchun, China.; 2 Jilin University College of Animal Science Laboratory Animal Center Changchun China Jilin University, College of Animal Science, Laboratory Animal Center, Changchun, China.; 3 Jilin University Hospital of Stomatology Jilin Provincial Key Laboratory of Tooth Development and Bone Remodeling Changchun China Jilin University, Hospital of Stomatology, Jilin Provincial Key Laboratory of Tooth Development and Bone Remodeling, Changchun, China.

**Keywords:** Ginsenoside Rd, H19 long non-coding RNA, E-cadherin, human, CRISPR-Cas Systems, Oral squamous cell carcinoma

## Abstract

**Objective:**

Tongue squamous cell carcinoma (TSCC) is an oral cancer, with high malignancy and frequent early migration and invasion. Only a few drugs can treat tongue cancer. Ginsenoside Rd is a ginseng extract with anti-cancer effects. Many noncoding RNAs are abnormally expressed in tongue cancer, thus influencing its occurrence and development. H19 and miR-675-5p can promote cancer cell growth. This study aimed to analyze the regulation effect of ginsenoside Rd on H19 and miR-675-5p in tongue cancer.

**Methodology:**

We used CCK8 and flow cytometry to study the growth and apoptosis. Transwell assay was used to assess invasion; wound-healing assay to assess migration; and colony formation assays to test the ability of cells to form colonies. H19, miR-675-5p, and CDH1 expressions were analyzed by qPCR. E-cadherin expression was detected using western blot. CRISPR/cas9 system was used for CDH1 knockout.

**Results:**

Ginsenoside Rd inhibited the growth and increased the apoptosis of SCC9 cells. Ginsenoside Rd also inhibited the migration and invasion of SCC9 cells. H19 and miR-675-5p were highly expressed, while CDH1 and E-cadherin expressions were low. H19 and miR-675-5p promoted SCC9 metastasis. In contrast, CDH1 and E-cadherin inhibited the metastasis of SCC9 cells. Bioinformatics analysis showed that miR-675-5p was associated with CDH1. H19 and miR-675-5p expressions decreased after ginsenoside Rd treatment, while CDH1 and E-cadherin expressions increased.

**Conclusions:**

Ginsenoside Rd inhibits tongue cancer cell migration and invasion via the H19/miR-675-5p/CDH1 axis.

## Introduction

Tongue squamous cell carcinoma (TSCC) is an oral cancer with high malignancy and frequent early migration and invasion.^1,2^ Smoking and drinking are the main etiologic factors of TSCC.^3^ Although surgical treatment has improved patients’ survival rate, it remains low.^4^ Moreover, surgery significantly damages patients’ body.^5^ Therefore, sometimes it is necessary to administer radiotherapy and chemotherapy to prevent cancer recurrence.^6,7^ Therefore, studying some drugs with low-damaging effect and gene therapy can enhance cancer prevention and treatment.

Ginseng is a famous nutrient and Chinese herbal medicine.^8^ High concentrations of ginsenoside, including ginsenoside Rb1, Rb2, Rc, Rd, and Re are found in ginseng.^9^ Ginsenoside Rd has anti-inflammatory, anti-aging, and nerve protection effects.^10-12^ Increasing evidence has also shown that ginseng and its purified ginsenosides have anti-cancer effects.^13^ Studies have also shown that diol-type ginsenosides have stronger anti-cancer activity than triol-type ginsenosides. Ginsenoside Rd has the strongest anti-cancer activity among the diol-type ginsenosides.^9,14,15^ Therefore, ginsenoside Rd can inhibit tongue cancer.

A recent study showed that ginsenoside Rb3 can regulate H19,^16^ indicating that ginsenoside Rd can inhibit tongue cancer by regulating H19. Noncoding RNA H19 is highly expressed in various cancers.^17^ For instance, miR-675-5p, a micro-RNA from H19, can bind to some specific mRNA-encoding proteins, thus inhibiting its expression.^18^ E-cadherin is closely related to cell adhesion and contact inhibition.^19^ The abnormal expression of E-cadherin in cancers may promote cancer metastasis.^20^ E-cadherin expression is decreased in oral cancers.^21^ Therefore, the irregular expression of E-cadherin can be used as a biomarker for clinical diagnosis, treatment, and prognosis of tumors,^22^ indicating that H19/miR-675-5p and CDH1 may be related.^23^

CRISPR/Cas9 is found in bacteria and archaea. It is mainly composed of three different types of systems, of which *Streptococcus thermophilus* or *Streptococcus pyogenes* type II system is the most widely used. Type II system is based on a single-guide RNA (sgRNA) and a cas9 protein that targets a sequence of DNA for editing. The CRISPR/Cas9 system has been widely used in cancer characterization and modeling in recent years and is promising in cancer treatment.^24^ For example, the tumor-targeting delivery system CRISPR/cas9 can downregulate hypoxia-inducible factor-1α (HIF-1α) *in vivo*, thus inhibiting the metastasis of pancreatic cancer.^25^

Although ginsenoside can inhibit many cancers, only a few studies have reported the inhibition effect of ginsenoside Rd on tongue cancer and its mechanism.^26-28^ This study aimed to assess the effect of ginsenoside Rd on tongue cancer cell migration and invasion and its mechanism.

## Methodology

### Tongue Cancer Tissues

The TSCC tissues (n=3) were taken from surgical patients at the Hospital of Stomatology of Jilin University (No. 2 in 2022) and stored at -80°C.

### Cell Culture

SCC9 and CAL27 cells (Atcc) were cultured in DMEM/F12 containing 10% FBS and 1% penicillin/streptomycin (Gibco) in an incubator with 5% carbon dioxide at 37°C. The 100 µM Rd (Meilunbio, China) was used to treat cells for qPCR, WB, apoptosis, migration, invasion and colony formation analyses.

### Overexpression and knockdown of miR-675-5p and H19

pcDNA3.1-H19, a plasmid for H19 overexpression, was sourced from the GenePharma (Shanghai, China). si-H19, a small interfering RNA that reduces H19 expression, was obtained from RiboBio. miR-675-5p inhibitors and mimics, which can reduce or overexpress miR-675-5p, were obtained from RiboBio.

### Overexpression and knockout of CDH1

pcDNA3.1-CDH1, a plasmid for CDH1 overexpression, was sourced from Sangon Biotech. CRISPR/cas9 system was used for CDH1 knockout.^29^ The px459 (Addgene, USA) was used as a knockout plasmid. sgRNA sequence: 5’-AAGTCACGCTGAATACAGTG-3’.^30^

### Transfection procedure

SCC9 cells were seeded in six-well plates (3×10^5^/well). Transfection was conducted when the cell density reached about 70% after culturing for 12-24 h. First, the medium without FBS was mixed with Lipofectamine 2000 and incubated for 5 minutes. The sample was then mixed with DNA (pcDNA3.1-H19, pcDNA3.1-CDH1, px459-CDH1) or miRNA (miR-675-5p mimics or inhibitors) diluted in a medium without FBS and incubated for 20 minutes. The mixture was then added to the cells, gently shaken to mix, and put in an incubator. The medium containing FBS was changed after 6 h, then cultured for 24-48 h.

### Quantitative real-time PCR

**Figure S1:**
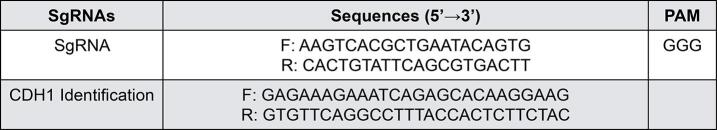
Sequence of sgRNA

**Figure S2:**
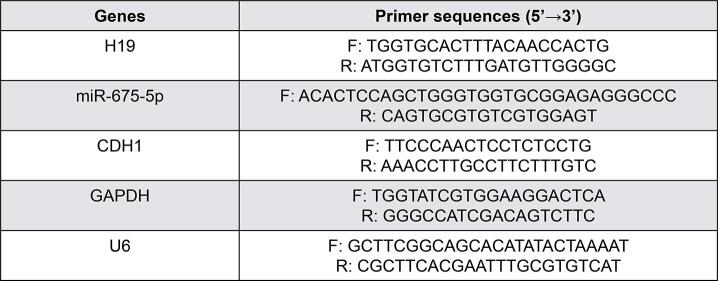
Primers for qPCR analysis

RNA was extracted from tongue cancer tissues and cells (SCC9) using TRIzol (Invitrogen, USA). A first-strand cDNA synthesis kit (Tiangen, China) was used to reverse-transcribe the RNA to obtain cDNA. SYBR Real-Time PCR kit (Tiangen, China) was used for the subsequent qPCR reaction of cDNA. qPCR conditions were: initial 94°C for 15 minutes, followed by 35 cycles of denaturation at 94°C for 15 seconds, annealing at 60°C for 30 seconds, and extension at 72°C for 20 seconds. GAPDH was used as an internal reference gene for H19 and CDH1. U6 was used as an internal reference gene for miR-675-5p. The primer sequence is listed in Figure S1 and Figure S2.

### Western blot

The protein extraction buffer (Beyotime, China) was used to extract the protein from the tongue cancer tissues and cells. A BCA kit (Meilunbio, China) was used to measure protein concentration. Notably, 10% SDS-PAGE gels were used to isolate proteins with different molecular weights when the protein concentration of the experimental and the control groups was the same. Next, the PVDF membrane was used to transfer the isolated proteins. The PVDF membranes were blocked with 5% non-fat milk powder for 1 h, then incubated with antibodies against E-cadherin (Proteintech, USA) and GAPDH (Bioworld, USA) at 4°C overnight. The PVDF membrane was then washed with TBST and incubated with secondary antibodies (Boster, China) for 2 h. ECL Super Signal (Thermo Fisher Scientific, USA) was used to detect the protein bands. ImageJ software was used for quantitative analysis of protein bands.

### Wound healing assay

SCC9 cells were seeded in six-well plates (3×10^5^/well). After transfection, a scratch was made while culturing the cells with a serum-free medium Images were obtained at 0, 24 and 48 h using an inverted microscope. ImageJ software was used to investigate the results.

### Transwell assay

In transwell assay, the upper chamber (Corning, USA) was filled with 20 μL of Matrigel (BD Biosciences, USA), then incubated at 37°C under 5% CO_2_ for 30 minutes. SCC9 cells (1×10^4^) were seeded, then transfected with si-H19, pcDNA3.1-H19, miR-675-5p inhibitors, miR-675-5p mimics, pcDNA3.1-CDH1, px459-CDH1, or treated with ginsenoside Rd (100 µM), and added into the upper chamber. Medium (0.5 mL) with 10% fetal bovine serum was added to the bottom chamber. The sample was cultured at 37°C under 5% CO_2_ for 48 h. Finally, an inverted microscope was used to visualize the cells at the bottom chamber after fixing with 4% paraformaldehyde. The cells were stained with 0.1% crystal violet dye (Solarbio, China). The stained cells were counted using Image J.

### Colony formation assay

SCC9 cells were seeded in 60-mm plates (1×10^3^) for two weeks. The cells were fixed with 4% paraformaldehyde and then stained with 0.1% crystal violet dye (Solarbio, China). Images were taken, and the cells were counted using Image J.

### Cell counting Kit-8 assay

The CCK8 assay (Meilunbio, China) was used to examine cell viability. Cells transfected with si-H19, pcDNA3.1-H19, miR-675-5p inhibitors, miR-675-5p mimics, pcDNA3.1-CDH1, px459-CDH1, or treated with ginsenoside Rd (100µM), were seeded into a 96-well plate for 24 h. CCK8 solution (10 μL) was then added to each well, then cultured at 37 °C under 5% CO_2_ for 2 h. Finally, absorbance was detected at 450 nm. The data were analyzed using GraphPad.

### Cell apoptosis analysis

Cells were first treated with ginsenoside Rd. The cells were then cultured at 37°C for 24 h under 5% CO_2_. An apoptosis kit (Beyotime, China) was used to treat cells following the instructions. Flow cytometry (BD Biosciences, USA) was used to measure apoptosis.

### Statistical analysis

Student’s t-tests were used to determine differences between groups. The data are expressed as mean±SD. SPSS 16.0 (SPSS Inc., USA) was used for all statistical analyses. P<0.05 indicated statistical significance.

## Results

### Ginsenoside Rd inhibits the growth, apoptosis, migration, and invasion of TSCC

**Figure 1:**
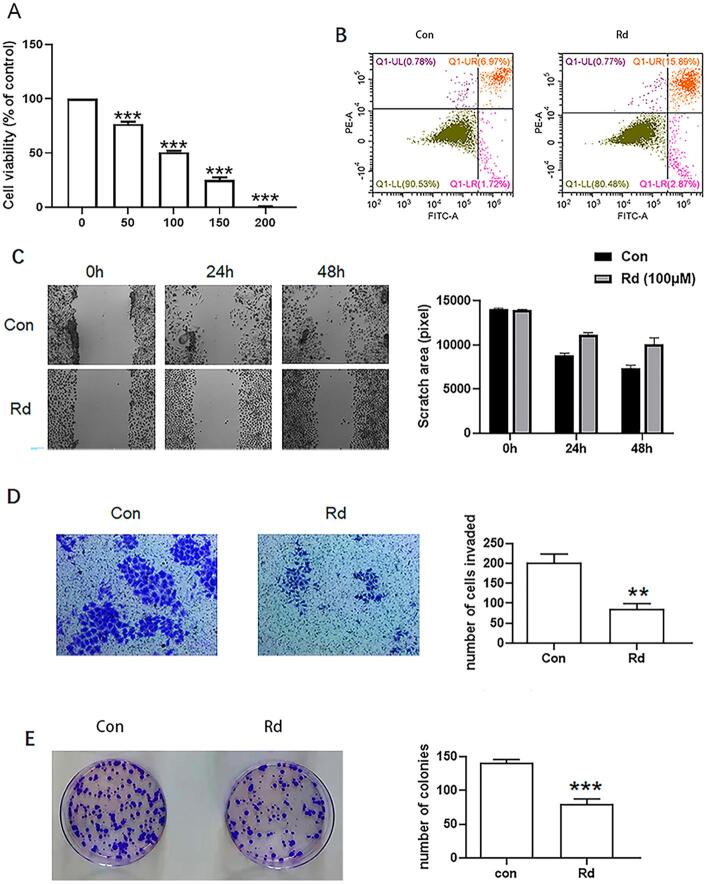
Effects of ginsenoside Rd on SCC9. (A) CCK8 showing the inhibitory effect of ginsenoside on SSC9. (B) Apoptosis rate of SCC9 after ginsenoside Rd treatment. (C) Migration of SCC9 after ginsenoside Rd treatment. (D) Invasion of SCC9 after ginsenoside Rd treatment. (E) colony formation of SCC9 after ginsenoside Rd treatment. Different letters indicate statistically significant differences in the mean±SD (n=3) (*P<0.05, **P<0.01, ***P<0.001, ****P<0.0001)

**Figure S3:**
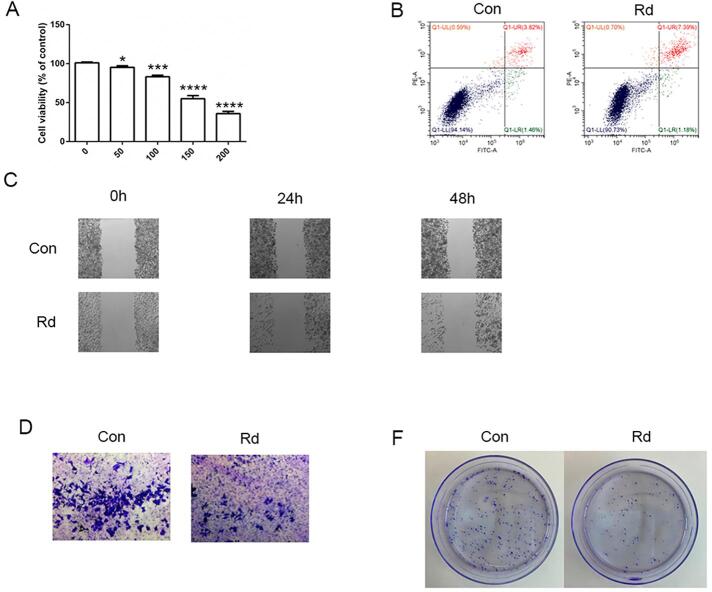
Effects of ginsenoside Rd on CAL27. (A) CCK8 showing the inhibitory effect of ginsenoside on CAL27. (B) Apoptosis rate of CAL27 after ginsenoside Rd treatment. (C) Migration of CAL27 after ginsenoside Rd treatment. (D) Invasion of CAL27 after ginsenoside Rd treatment. (E) Colony formation of CAL27 after ginsenoside Rd treatment. Different letters indicate statistically significant differences in the mean±SD (n=3) (*P<0.05, **P<0.01, ***P<0.001, ****P<0.0001).

CCK8 showed that cell growth was significantly inhibited when the SCC9 was treated with 100 μM ginsenoside Rd (Figure 1A). The cell activity was negatively correlated with the ginsenoside Rd concentration. Flow cytometry analysis (Figure 1B) showed that ginsenoside Rd increased the SCC9 apoptosis. Wound healing assays and Transwell (Figure 1C, D) showed that ginsenoside Rd inhibited the migration and invasion of SCC9. The colony formation assay (Figure 1E) showed SCC9 clone was decreased after ginsenoside Rd treatment. Moreover, 100 μM Rd inhibited the growth, migration and invasion of CAL27 cells, inducing apoptosis (Figure S3). These results show that ginsenoside Rd can prevent the growth, invasion, and migration of TSCC cells and promote apoptosis.

### H19 and miR-675-5p promote TSCC metastasis

**Figure 2:**
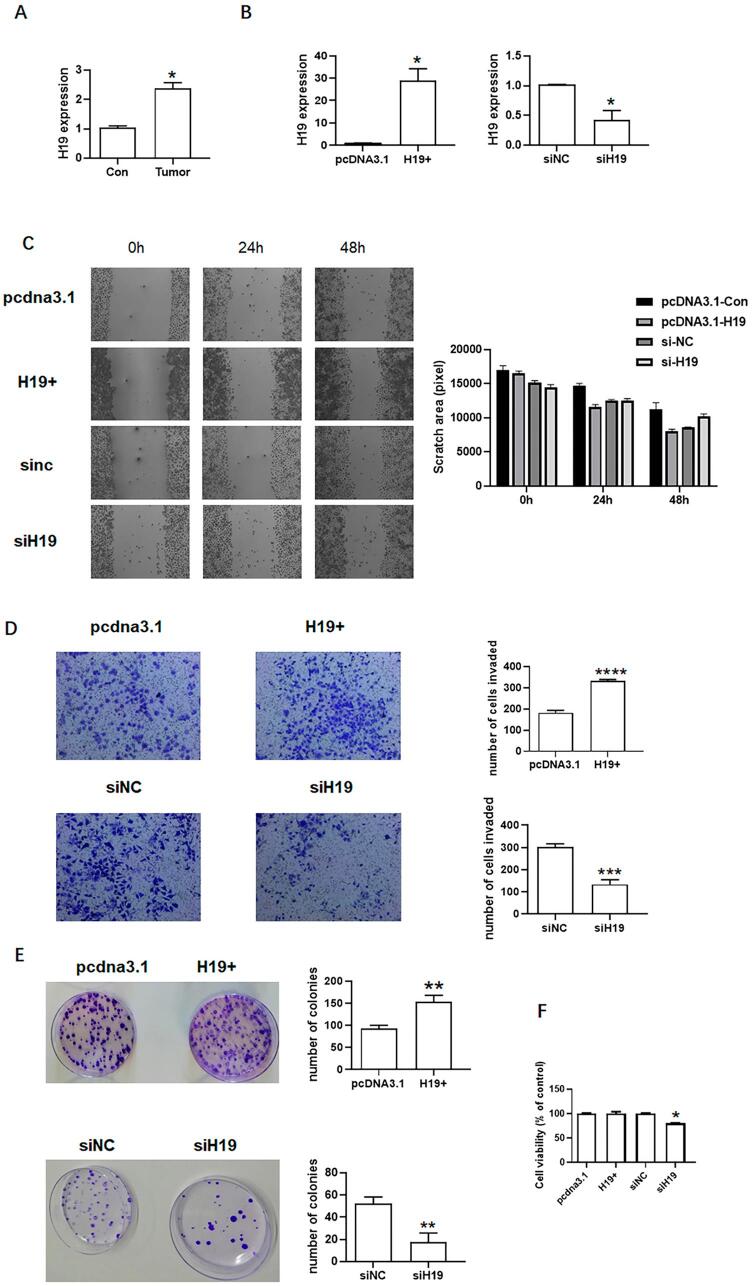
Effects of H19 on SCC9. (A) H19 expression in tongue cancer (n=3). (B) Overexpression and knockdown of H19. (C) Migration of SCC9 after H19 overexpression and knockdown. (D) Invasion of SCC9 after H19 overexpression and knockdown. (E) Colony formation of SCC9 after H19 overexpression and knockdown. (F) Growth of SCC9 after H19 overexpression and knockdown. Different letters indicate statistically significant differences in the mean±SD (n=3) (*P<0.05, **P<0.01, ***P<0.001, ****P<0.0001).

**Figure 3:**
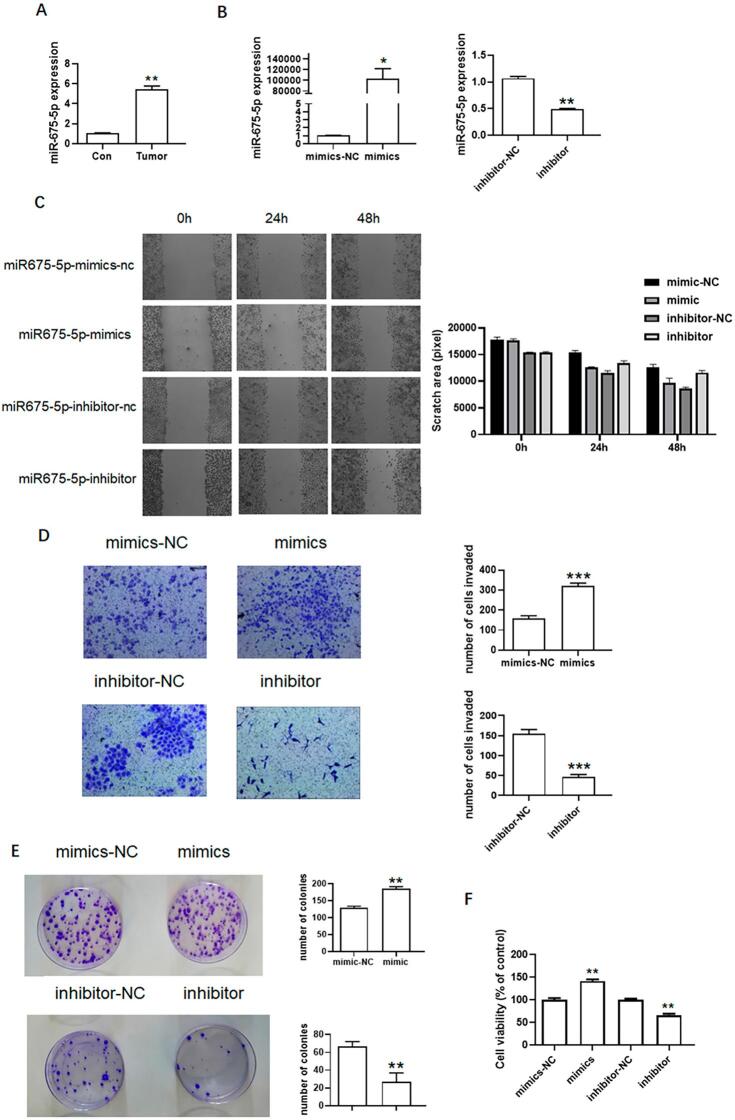
Effects of miR-675-5p on SCC9. (A) miR-675-5p expression in tongue cancer cells (n=3). (B) Overexpression and knockdown of miR-675-5p. (C) Migration of SCC9 after miR-675-5p overexpression and knockdown. (D) Invasion of SCC9 after miR-675-5p overexpression and knockdown. (E) Colony formation of SCC9 after miR-675-5p overexpression and knockdown. (F) Growth of SCC9 after miR-675-5p overexpression and knockdown. Different letters indicate statistically significant differences in the mean±SD (n=3) (*P<0.05, **P<0.01, ***P<0.001, ****P<0.0001).

Previous studies showed that H19 promotes the metastasis of cancer cells, including colorectal cancers.^31^ This study aimed to determine if H19 is associated with tongue cancer. qPCR results showed that H19 expression was higher in tongue cancer tissues than in normal tissues (Figure 2A). We overexpressed and knocked down H19 (Figure 2B) in SCC9 for further analysis. Compared to the control group, H19 overexpression enhanced the migration ability of SCC9, while H19 knockdown inhibited migration (Figure 2C). Moreover, H19 overexpression enhanced the invasion ability of SCC9, while H19 knockdown inhibited invasion (Figure 2D). The colony formation assay (Figure 2E) showed that H19 overexpression increased the number of SCC9 clones, while H19 knockdown decreased the number of SCC9 clones. Although H19 overexpression did not significantly affect the growth ability of SCC9, H19 knockdown inhibited proliferation of SCC9 (Figure 2F). qPCR analyses showed that miR-675-5p expression was higher in tongue cancer tissues than in normal tissues (Figure 3A). Furthermore, miR-675-5p overexpression and knockdown analyses were conducted in SCC9 to assess their effects on migration, invasion, growth, and the number of clones (Fig. 3B). miR-675-5p overexpression increased the ability of migration, invasion, growth, and number of clones of SCC9. In contrast, miR-675-5p knockdown decreased the migration, invasion, growth and number of clones (Figures 3C, D, E, F). These results indicate that H19 and miR-675-5p can promote metastasis of tongue cancer.

### E-cadherin inhibits metastasis of tongue cancer

**Figure 4:**
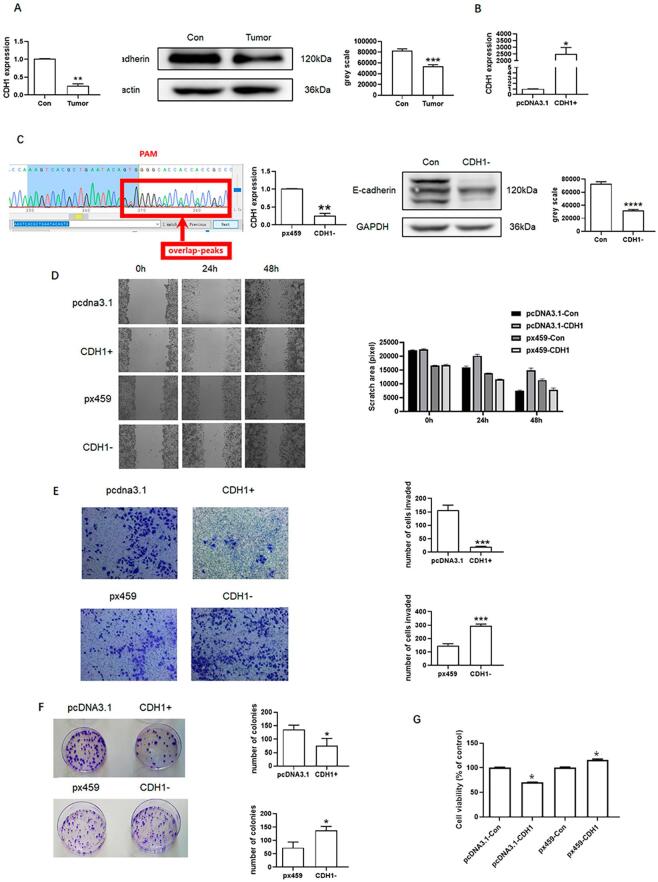
Effects of CDH1 on SCC9. (A) CDH1 and E-cadherin expressions in tongue cancer. (B) Overexpression of CDH1. (C) Knockdown of CDH1. (D) Migration of SCC9 after CDH1 overexpression and knockdown. (E) Invasion of SCC9 after CDH1 overexpression and knockdown. (F) Colony formation of SCC9 after CDH1 overexpression and knockdown. (G) Growth of SCC9 after CDH1 overexpression and knockdown. Different letters indicate statistically significant differences in the mean±SD (n=3) (*P<0.05, **P<0.01, *** P<0.001, ****P<0.0001).

E-cadherin regulates cancer metastasis.^32^ In this study, qPCR showed that CDH1 expression was lower in tongue cancer tissue than in normal tongue tissue. Western blotting also showed decreased expression of E-cadherin in tongue cancer tissue (Figure 4A). Therefore, CDH1 was overexpressed to further study the relationship between E-cadherin and metastasis of tongue cancer (Figure 4B). CDH1 overexpression decreased the invasion, migration, growth and number of clones (Figures 4D-G) in tongue cancer cells. Finally, CRISPR/Cas9 system was used to knock down CDH1 to further assess the relationship (Figure 4C). Compared to the control group, CDH1 knockdown increased the migration, invasion, growth and number of clones (Figures 4D-G) of SCC9, indicating that E-cadherin is closely associated with the metastasis of tongue cancer.

### Mechanism of ginsenoside Rd inhibition of migration and invasion of tongue cancer cells

**Figure 5:**
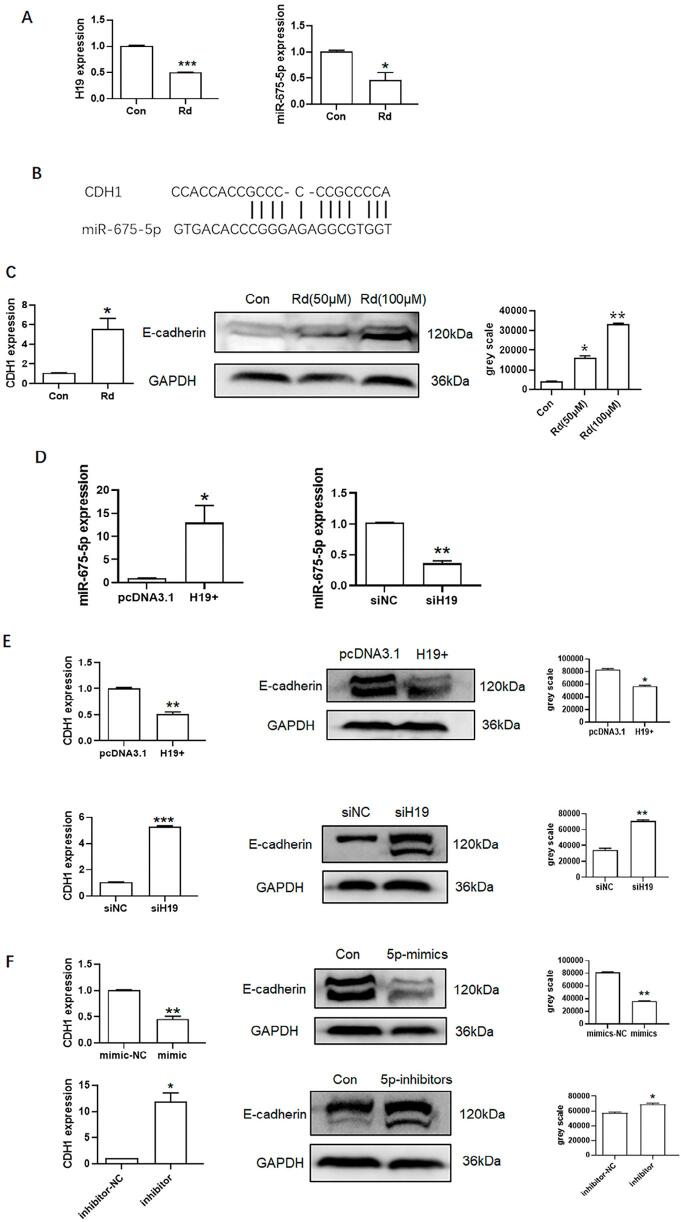
Mechanism of inhibition effect of ginsenoside Rd on migration and invasion of SCC9. (A) H19 and miR-675-5p expressions in SCC9 after ginsenoside Rd treatment. (B) The binding site of CDH1 for miR-675-5p. (C) CDH1 and E-cadherin expressions in SCC9 after ginsenoside Rd treatment. (D) miR-675-5p expression after H19 overexpression and knockdown. (E) CDH1 and E-cadherin expressions after H19 overexpression and knockdown. (F) CDH1 and E-cadherin expressions after miR-675-5p overexpression and knockdown. Different letters indicate statistically significant differences in the mean±SD (n = 3) (*P<0.05, **P<0.01, ***P<0.001, ****P<0.0001).

**Figure S4:**
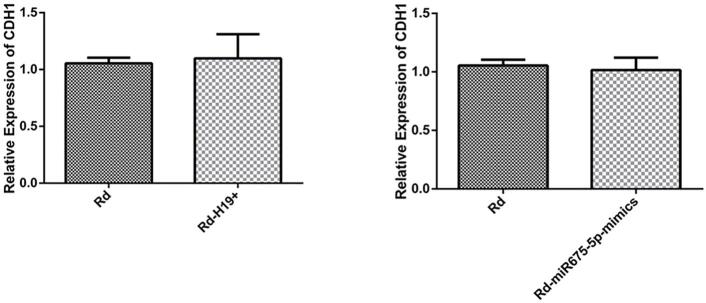
The expression of CDH1 after the overexpression of H19 and miR-675-5p with ginsenoside Rd treatment

Herein, H19 and miR-675-5p expression decreased after ginsenoside Rd treatment (Figure 5A). The bioinformatics method (https://cm.jefferson.edu/rna22/) showed that miR-675-5p negatively regulated CDH1 (Figure 5B). Moreover, qPCR showed that CDH1 expression increased. Western blotting also showed that E-cadherin expression increased (Figure 5C) after ginsenoside Rd treatment. H19 overexpression increased miR-675-5p expression while H19 knockdown decreased miR-675-5p expression (Figure 5D). H19 overexpression also decreased CDH1 and E-cadherin expressions, while H19 knockdown increased CDH1 and E-cadherin expressions (Figure 5E). miR-675-5p overexpression decreased CDH1 and E-cadherin expression, while miR-675-5p knockdown increased CDH1 and E-cadherin expressions (Fig. 5F). The overexpression of H19 and miR-675-5p can alleviate the effect of ginsenoside Rd treatment on the expression of CDH1 (Figure S4). These results indicate that ginsenoside Rd prevents the invasion and migration of SCC9 through H19/miR-675-5p/CDH1 axis.

## Discussion

Ginsenoside Rd is an extract of ginseng with anti-tumor, anti-inflammatory, and nerve protection effects.^10,11,13^ Ginsenoside Rd can inhibit colorectal cancer.^33^ However, only a few studies have reported on the effect of ginsenoside Rd on tongue cancer. In this study, ginsenoside Rd suppressed the growth, invasion and migration of SCC9 while it promoted apoptosis. Therefore, further studies should assess the mechanism of inhibition effect of ginsenoside Rd on migration and invasion of tongue cancer cells.

Our study found that ginsenoside Rd regulated the expression of H19. H19 is a long noncoding RNA that cannot form proteins and the embryonic expression of H19 is suppressed after birth.^34^ H19 is related to many cancers. For instance, H19 enhances autophagy in estrogen receptor-positive breast cancer cells by reducing the methylation of the Beclin1 promoter region via the H19/SAHH/DNMT3B axis, leading to drug resistance.^35^ H19 promotes cancer development mainly by affecting cell functions, such as cell proliferation, anti-apoptosis, and thus leading to angiogenesis and immune escape.^36^

In this study, H19 expression was high in tongue cancer and promoted the invasion and migration of tongue cancer cells. Some previous experiments confirmed that H19 promotes TSCC migration and invasion,^37^ consistent with this research. Furthermore, miR-675 is found in H19. Peperstraete, et al. (2020) found that miR-675 and H19 are involved in cancer migration and invasion alone or together.^38^

In this study, H19, as a functional RNA, acted as a regulator.^39^ Bioinformatics methods showed that miR-675-5p has binding sites for the mRNA of CDH1, affecting the E-cadherin expression. Moreover, overexpression of E-cadherin inhibited migration and invasion of tongue cancer cells. CDH1 knockdown increased the migration and invasion ability of tongue cancer cells. Some researchers found that samples with lymph node metastasis have lower E-cadherin expression.^40^ Some scholars have confirmed that E-cadherin is a promising biomarker of TSCC.^41^ Therefore, H19/miR-675-5p/CDH1 is a key signaling pathway in the migration and invasion of tongue cancer cells.

## Conclusions

**Figure S5:**
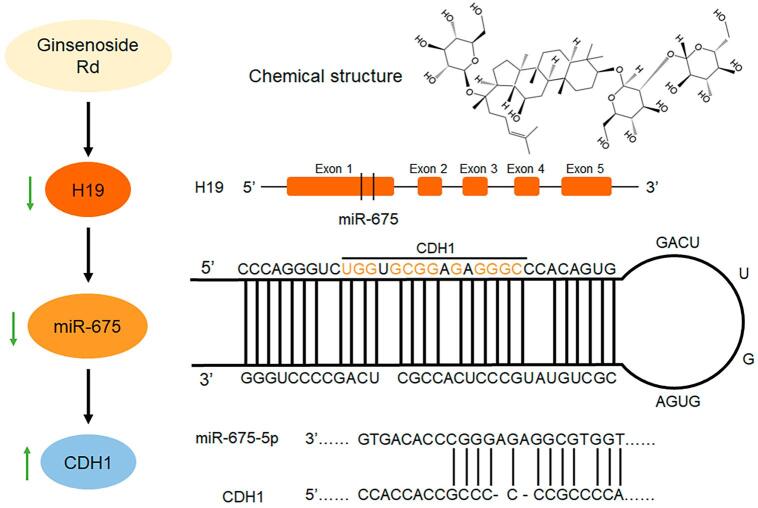
Schematic diagram of ginsenoside Rd interacting with the H19/miR-675-5p/CDH1 axis

In short, ginsenoside Rd inhibits migration and invasion of TSCC cells. Ginsenoside Rd can also regulate the migration and invasion of tongue cancer cells through the H19/miR-675-5p/CDH1 axis. Therefore, this research shows that ginsenoside Rd may be a natural anti-cancer drug. Moreover, H19 and CDH1 may be potential markers of tongue cancer. (Figure S5).

## References

[1] Shetty SS, Kudpaje A, Jayaraj R, Rao V, Shah PK. Tongue cancer: a discrete oral cavity subsite. Oral Oncol. 2019;99:104348. doi: 10.1016/j.oraloncology.2019.06.029 10.1016/j.oraloncology.2019.06.029 31272801

[2] Amini A. Early stage oral tongue cancer with an ipsilateral nodal recurrence 2 years later: what do you treat? Int J Radiat Oncol. 2020;106(5):900-1. doi: 10.1016/j.ijrobp.2018.12.035 10.1016/j.ijrobp.2018.12.035 32171457

[3] Miranda-Filho A, Bray F. Global patterns and trends in cancers of the lip, tongue and mouth. Oral Oncol. 2020;102:104551. doi: 10.1016/j.oraloncology.2019.104551 10.1016/j.oraloncology.2019.104551 31986342

[4] Lee DY, Kang SH, Kim JH, Kim MS, Oh KH, Woo JS, et al. Survival and recurrence of resectable tongue cancer: resection margin cutoff value by T classification. Head Neck. 2018;40(2):283-91. doi: 10.1002/hed.24944 10.1002/hed.24944 28960654

[5] Hancock SB, Krempl GA, Canfield V, Bogardus C, Kojouri K, Kaneaster SK, et al. Treatment of base of tongue cancer with paclitaxel, ifosfamide, and cisplatinum induction chemotherapy followed by chemoradiotherapy. Laryngoscope. 2008;118(8):1357-61. doi: 10.1097/MLG.0b013e318175336a 10.1097/MLG.0b013e318175336a 18528311

[6] So YK, Oh D, Choi N, Baek CH, Ahn YC, Chung MK. Efficacy of postoperative neck irradiation for regional control in patients with pN0 oral tongue cancer: propensity analysis. Head Neck-J Sci Spec. 2018;40(1):163-9. doi: 10.1002/hed.24980 10.1002/hed.24980 29083541

[7] Park S, Cho Y, Lee J, Koh YW, Kim SH, Choi EC, et al. Survival and functional outcome after treatment for primary base of tongue cancer: a comparison of definitive chemoradiotherapy versus surgery followed by adjuvant radiotherapy. Cancer Res Treat. 2018;50(4):1214-25. doi: 10.4143/crt.2017.498 10.4143/crt.2017.498 PMC6192900 29281874

[8] Hsu BY, Lu TJ, Chen CH, Wang SJ, Hwang LS. Biotransformation of ginsenoside Rd in the ginseng extraction residue by fermentation with lingzhi (Ganoderma lucidum). Food Chem. 2013;141(4):4186-93. doi: 10.1016/j.foodchem.2013.06.134 10.1016/j.foodchem.2013.06.134 23993604

[9] Kim YJ, Yamabe N, Choi P, Lee JW, Ham J, Kang KS. Efficient thermal deglycosylation of ginsenoside rd and its contribution to the improved anticancer activity of ginseng. J Agr Food Chem. 2013;61(38):9185-91. doi: 10.1021/jf402774d 10.1021/jf402774d 23984628

[10] Wu SD, Xia F, Lin XM, Duan KL, Wang F, Lu QL, et al. Ginsenoside-Rd promotes neurite outgrowth of pc12 cells through MAPK/ERK- and PI3K/AKT-Dependent Pathways. Int J Mol Sci. 2016;177. doi: 10.3390/ijms17020177 10.3390/ijms17020177 PMC4783911 26840295

[11] Zhang YX, Wang L, Xiao EL, Li SJ, Chen JJ, Gao B, et al. Ginsenoside-Rd exhibits anti-inflammatory activities through elevation of antioxidant enzyme activities and inhibition of JNK and ERK activation *in vivo*. Int Immunopharmacol. 2013;17(4):1094-100. doi: 10.1016/j.intimp.2013.10.013 10.1016/j.intimp.2013.10.013 24455777

[12] Yu XX, Li H, Lin DF, Guo WZ, Xu ZH, Wang LP, et al. Ginsenoside prolongs the lifespan of c. elegans via lipid metabolism and activating the stress response signaling pathway. Int J Mol Sci. 2021;22(18):9668. doi: 10.3390/ijms22189668 10.3390/ijms22189668 PMC8465798 34575832

[13] Tian YZ, Liu YP, Tian SC, Ge SY, Wu YJ, Zhang BL. Antitumor activity of ginsenoside Rd in gastric cancer via up-regulation of Caspase-3 and Caspase-9. Pharmazie. 2020;75(4):147-50. doi: 10.1691/ph.2020.9931 10.1691/ph.2020.9931 32295691

[14] Zhong CJ, Jiang C, Ni SY, Wang QZ, Cheng LG, Wang H, et al. Identification of bioactive anti-angiogenic components targeting tumor endothelial cells in Shenmai injection using multidimensional pharmacokinetics. Acta Pharmacol Sin B. 2020;10(9):1694-708. doi: 10.1016/j.apsb.2019.12.011 10.1016/j.apsb.2019.12.011 PMC7564034 33088689

[15] Chang TL, Ding HY, Kao YW. Role of Ginsenoside Rd in Inhibiting 26S proteasome activity. J Agr Food Chem. 2008;56(24):12011-5. doi: 10.1021/jf801427e 10.1021/jf801427e 19053398

[16] Tan Y, Sun D, Chen J, Li R, Wang S. Ginsenoside Rb3 alleviates smoke-induced lung injury via the H19/miR-29b-3p/HGMB1/TLR4 signalling pathway. J Cell Mol Med. 2021;25(5):2725-9. doi: 10.1111/jcmm.15844 10.1111/jcmm.15844 PMC7933968 33523607

[17] Zhang Y, Huang W, Yuan Y, Li J, Wu J, Yu J, et al. Long non-coding RNA H19 promotes colorectal cancer metastasis via binding to hnRNPA2B1. J Exp Clin Cancer Res. 2020;39(1):141. doi: 10.1186/s13046-020-01619-6 10.1186/s13046-020-01619-6 PMC7412843 32698890

[18] Chen S, Bu D, Ma Y, Zhu J, Chen G, Sun L, et al. H19 overexpression induces resistance to 1,25(OH)2D3 by Targeting VDR through miR-675-5p in colon cancer cells. neoplasia. 2017;19(3):226-36. doi: 10.1016/j.neo.2016.10.007 10.1016/j.neo.2016.10.007 PMC5300698 28189050

[19] Biswas KH. Molecular mobility-mediated regulation of e-cadherin adhesion. Trends Biochem Sci. 2020;45(2):163-73. doi: 10.1016/j.tibs.2019.10.012 10.1016/j.tibs.2019.10.012 31810601

[20] Mendonsa AM, Na TY, Gumbiner BM. E-cadherin in contact inhibition and cancer. Oncogene. 2018;37(35):4769-80. doi: 10.1038/s41388-018-0304-2 10.1038/s41388-018-0304-2 PMC6119098 29780167

[21] Lopez-Verdin S, Martinez-Fierro ML, Garza-Veloz I, Zamora-Perez A, Grajeda-Cruz J, Gonzalez-Gonzalez R, et al. E-Cadherin gene expression in oral cancer: clinical and prospective data. Med Oral Patol Oral Cir Bucal. 2019;24(4):e444-e51. doi: 10.4317/medoral.23029 10.4317/medoral.23029 PMC6667017 31256188

[22] Wong SH, Fang CM, Chuah LH, Leong CO, Ngai SC. E-cadherin: its dysregulation in carcinogenesis and clinical implications. Crit Rev Oncol Hematol. 2018;121:11-22. doi: 10.1016/j.critrevonc.2017.11.010 10.1016/j.critrevonc.2017.11.010 29279096

[23] Luo M, Li Z, Wang W, Zeng Y, Liu Z, Qiu J. Long non-coding RNA H19 increases bladder cancer metastasis by associating with EZH2 and inhibiting E-cadherin expression. Cancer Lett. 2013;333(2):213-21. doi: 10.1016/j.canlet.2013.01.033 10.1016/j.canlet.2013.01.033 23354591

[24] Chira S, Gulei D, Hajitou A, Zimta AA, Cordelier P, Berindan-Neagoe I. CRISPR/Cas9: transcending the reality of genome editing. Mol Ther Nucleic Acids. 2017;7:211-22. doi: 10.1016/j.omtn.2017.04.001 10.1016/j.omtn.2017.04.001 PMC5415201 28624197

[25] Li M, Xie H, Liu Y, Xia C, Cun X, Long Y. Knockdown of hypoxia-inducible factor-1 alpha by tumor targeted delivery of CRISPR/Cas9 system suppressed the metastasis of pancreatic cancer. J Control Release. 2019;304:204-15. doi: 10.1016/j.jconrel.2019.05.019 10.1016/j.jconrel.2019.05.019 31100311

[26] Liang Y, Zhang TH, Jing SY, Zuo P, Li TZ, Wang YJ, et al. 20(S)-Ginsenoside Rg3 inhibits lung cancer cell proliferation by targeting EGFR-mediated Ras/Raf/MEK/ERK Pathway. Am J Chinese Med. 2021;49(03):753-65. doi: 10.1142/S0192415x2150035x 10.1142/S0192415X2150035X 33641655

[27] Ren ZG, Chen XM, Hong LJ, Zhao XX, Cui GY, Li A, et al. Nanoparticle conjugation of Ginsenoside Rg3 inhibits hepatocellular carcinoma development and metastasis. Small. 2020;16(2):e1905233. doi: 10.1002/smll.201905233 10.1002/smll.201905233 31814271

[28] Yin Q, Chen H, Ma RH, Zhang YY, Liu MM, Thakur K, et al. Ginsenoside CK induces apoptosis of human cervical cancer HeLa cells by regulating autophagy and endoplasmic reticulum stress. Food Funct. 2021;12(12):5301-16. doi: 10.1039/d1fo00348h 10.1039/d1fo00348h 34013944

[29] Shalem O, Sanjana NE, Hartenian E, Shi X, Scott DA, Mikkelson T, et al. Genome-scale CRISPR-Cas9 knockout screening in human cells. Science. 2014;343(6166):84-7. doi: 10.1126/science.1247005 10.1126/science.1247005 PMC4089965 24336571

[30] Wijshake T, Zou Z, Chen B, Zhong L, Xiao G, Xie Y, et al. Tumor-suppressor function of Beclin 1 in breast cancer cells requires E-cadherin. Proc Natl Acad Sci U S A. 2021;118(5):e2020478118. doi: 10.1073/pnas.2020478118 10.1073/pnas.2020478118 PMC7865132 33495338

[31] Rokavec M, Horst D, Hermeking H. Cellular model of colon cancer progression reveals signatures of mRNAs, miRNA, lncRNAs, and epigenetic modifications associated with metastasis. Cancer Res. 2017;77(8):1854-67. doi: 10.1158/0008-5472.CAN-16-3236 10.1158/0008-5472.CAN-16-3236 28130225

[32] Zhao T, Jiang W, Wang X, Wang H, Zheng C, Li Y, et al. ESE3 inhibits pancreatic cancer metastasis by upregulating E-Cadherin. Cancer Res. 2017;77(4):874-85. doi: 10.1158/0008-5472.CAN-16-2170 10.1158/0008-5472.CAN-16-2170 PMC5313376 27923832

[33] Phi LT, Sari IN, Wijaya YT, Kim KS, Park K, Cho AE, et al. Ginsenoside Rd inhibits the metastasis of colorectal cancer via epidermal growth factor receptor signaling axis. IUBMB Life. 2019;71(5):601-10. doi: 10.1002/iub.1984 10.1002/iub.1984 30576064

[34] Lecerf C, Le Bourhis X, Adriaenssens E. The long non-coding RNA H19: an active player with multiple facets to sustain the hallmarks of cancer. Cell Mol Life Sci. 2019;76(23):4673-87. doi: 10.1007/s00018-019-03240-z 10.1007/s00018-019-03240-z PMC11105575 31338555

[35] Wang J, Xie S, Yang J, Xiong H, Jia Y, Zhou Y, et al. The long noncoding RNA H19 promotes tamoxifen resistance in breast cancer via autophagy. J Hematol Oncol. 2019;12(1):81. Epub 20190724. doi: 10.1186/s13045-019-0747-0. 10.1186/s13045-019-0747-0 PMC6657081 31340867

[36] Fang Y, Fullwood MJ. Roles, functions, and mechanisms of long non-coding RNAs in cancer. Genomics Proteomics Bioinformatics. 2016;14(1):42-54. doi: 10.1016/j.gpb.2015.09.006 10.1016/j.gpb.2015.09.006 PMC4792843 26883671

[37] Kou N, Liu S, Li X, Li W, Zhong W, Gui L, et al. H19 facilitates tongue squamous cell carcinoma migration and invasion via sponging miR-let-7. Oncol Res. 2019;27(2):173-82. doi: 10.3727/096504018X15202945197589 10.3727/096504018X15202945197589 PMC7848458 29523225

[38] Peperstraete E, Lecerf C, Collette J, Vennin C, Raby L, Volkel P, et al. Enhancement of breast cancer cell aggressiveness by lncRNA H19 and its Mir-675 derivative: insight into shared and different actions. Cancers (Basel). 2020;12(7). doi: 10.3390/cancers12071730 10.3390/cancers12071730 PMC7407157 32610610

[39] Ali T, Grote P. Beyond the RNA-dependent function of LncRNA genes. Elife. 2020;9:e60583. doi: 10.7554/eLife.60583 10.7554/eLife.60583 PMC7584451 33095159

[40] Na TY, Schecterson L, Mendonsa AM, Gumbiner BM. The functional activity of E-cadherin controls tumor cell metastasis at multiple steps. Proc Natl Acad Sci U S A. 2020;117(11):5931-7. doi: 10.1073/pnas.1918167117 10.1073/pnas.1918167117 PMC7084067 32127478

[41] Hussein AA, Forouzanfar T, Bloemena E, Visscher JG, Brakenhoff RH, Leemans CR, et al. A review of the most promising biomarkers for early diagnosis and prognosis prediction of tongue squamous cell carcinoma. Brit J Cancer. 2018;119(6):724-36. doi: 10.1038/s41416-018-0233-4 10.1038/s41416-018-0233-4 PMC6173763 30131545

